# Supplementary material for: Effects of Probiotics on Gut Microbiomes of Extremely Preterm Infants in the Neonatal Intensive Care Unit: A Prospective Cohort Study
Source: Nutrients. 2022 Aug 8;14(15):3239. doi: 10.3390/nu14153239 (PMC9370381; doi:10.3390/nu14153239)
Supplement: Supplementary file 1 [file nutrients-14-03239-s001.zip › Supplemental Figure legends.pdf]

Effects of probiotics on gut microbiomes of extremely preterm infants in the neonatal intensive care unit: a prospective cohort study

Supplementary Figure Legends

Supplementary Figure S1. The Alpha Diversity Indexes of three subgroups (neonates with breast feeding, formula feeding and mixed feeding) are compared using the Observed, Chao1, ACE index, Shannon, Simpson InvSimpson, and Fisher Methods, which show no significant differences between these three subgroups.

Supplementary Figure S2. The PCoA was conducted to analyze the fecal microbiota composition in preterm infants administered with breast feeding (red color), formula feeding (green color), and mixed feeding (blue color) based on A) Bray-curtis distance, B) unweighted unifrac distance, and C) weighted unifrac distance.
