# Supplementary figures and images for: Effects of Probiotics on Gut Microbiomes of Extremely Preterm Infants in the Neonatal Intensive Care Unit: A Prospective Cohort Study
Source: Nutrients. 2022 Aug 8;14(15):3239. doi: 10.3390/nu14153239 (PMC9370381; doi:10.3390/nu14153239)

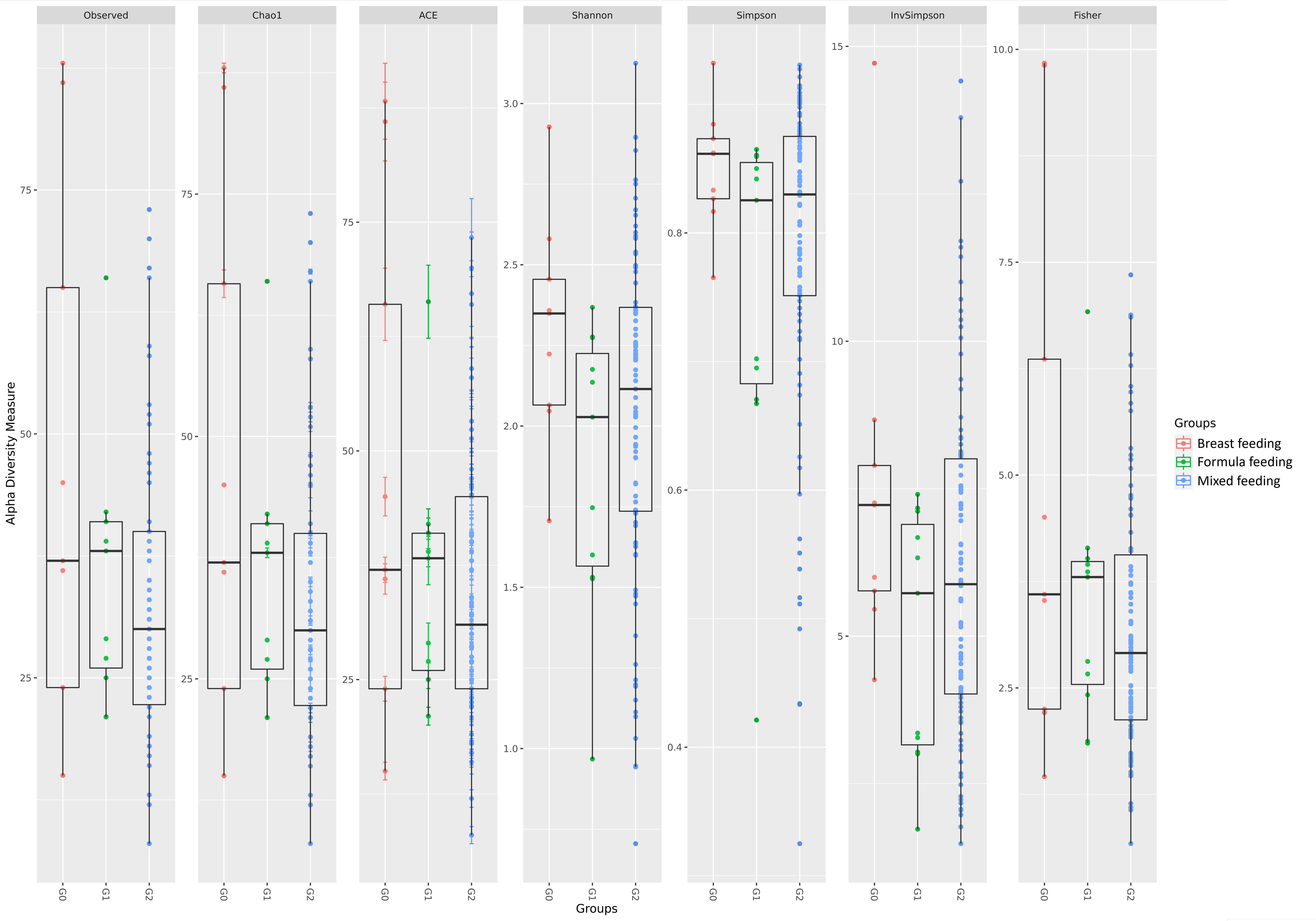

Supplement: Supplementary file 1 [file nutrients-14-03239-s001.zip › Supplemental Figure S1.pdf]

**A**

# Bray NMDS: Groups

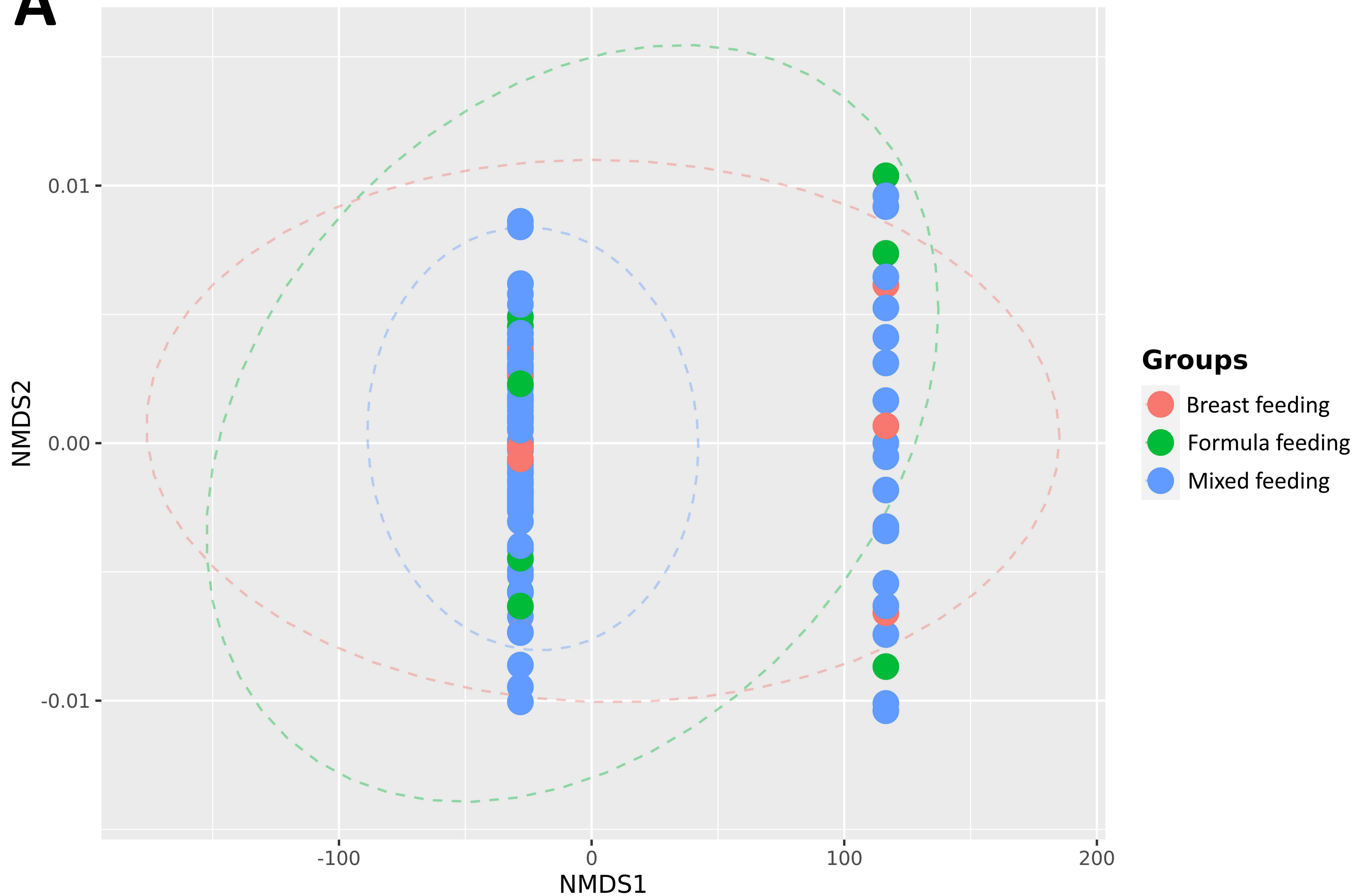

Supplement: Supplementary file 1 [file nutrients-14-03239-s001.zip › Supplemental Figure S2A.pdf]

**B** PCoA plot (the uweighted Unifrac distance): Groups

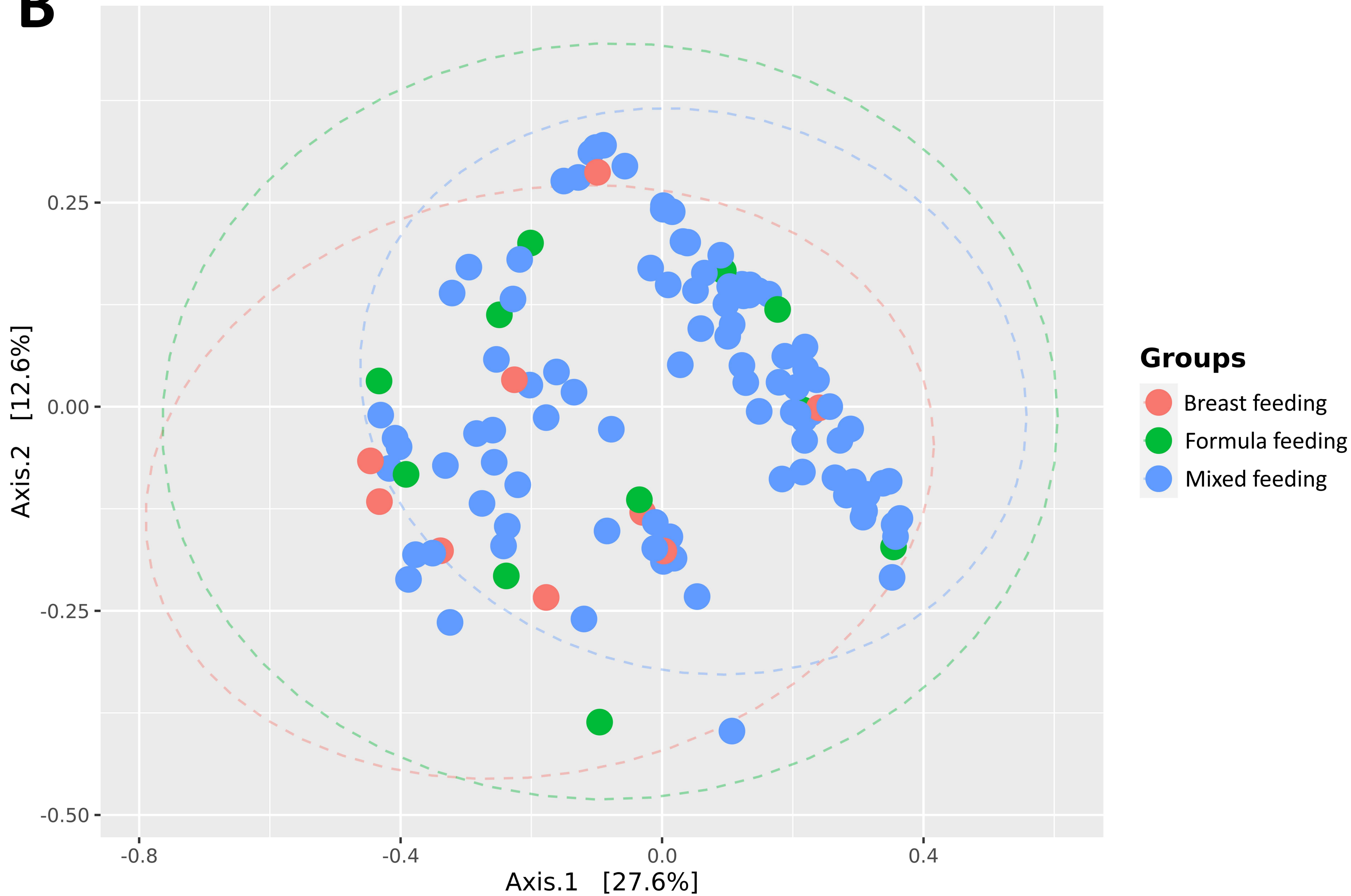

Supplement: Supplementary file 1 [file nutrients-14-03239-s001.zip › Supplemental Figure S2B.pdf]

**C** PCoA plot (the weighted Unifrac distance): Groups

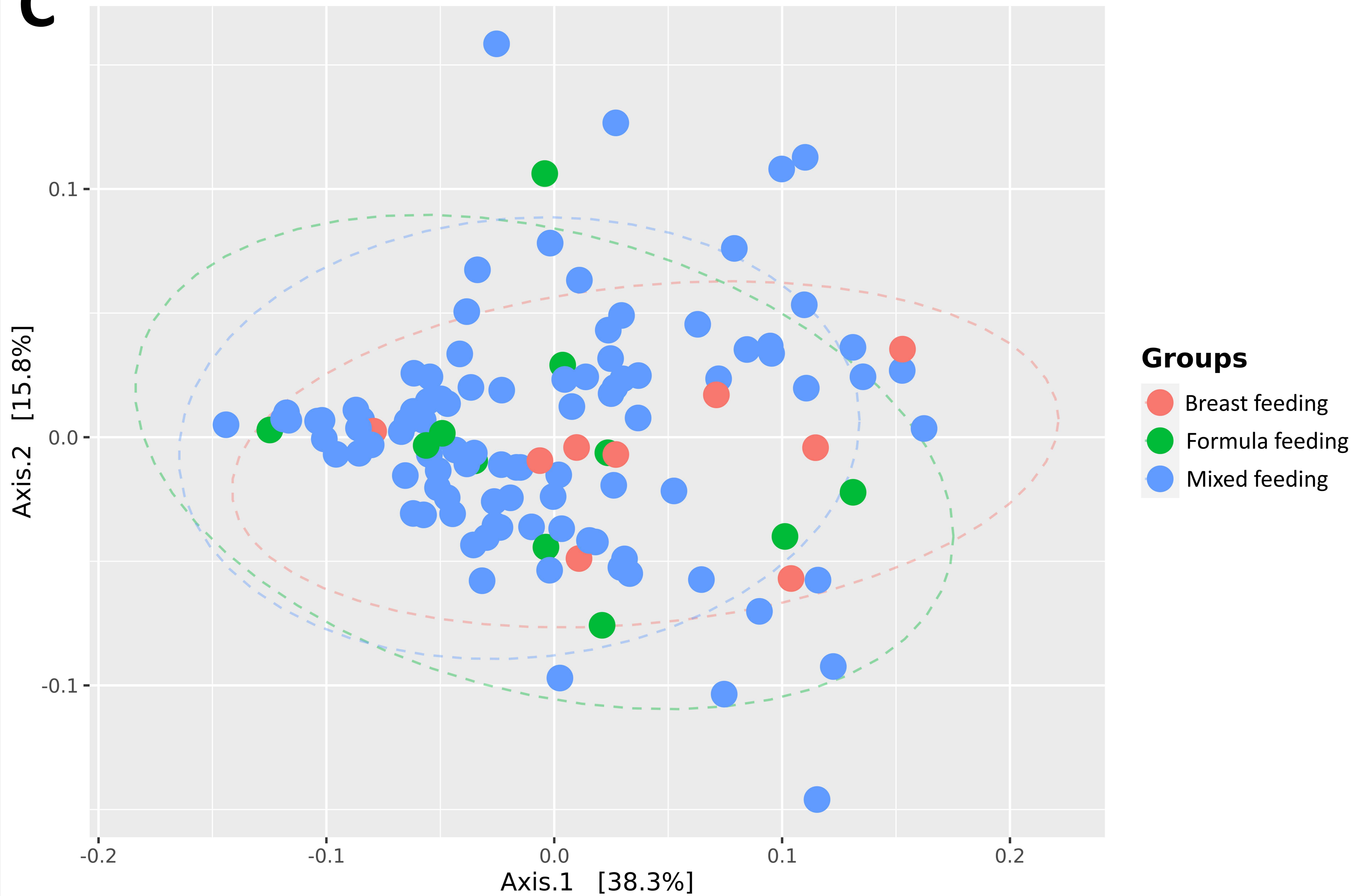

Supplement: Supplementary file 1 [file nutrients-14-03239-s001.zip › Supplemental Figure S2C.pdf]
